# Is mechanical power an under-recognised entity within the preterm lung?

**DOI:** 10.1186/s40635-023-00511-9

**Published:** 2023-05-22

**Authors:** David G. Tingay, Hannah Naidu, Hamish D. Tingay, Prue M. Pereira-Fantini, Martin C. J. Kneyber, Tobias Becher

**Affiliations:** 1 Neonatal Research, Murdoch Children’s Research Institute, Parkville, Australia; 2 Department of Paediatrics, University of Melbourne, Melbourne, Australia; 3 Department of Neonatology, The Royal Children’s Hospital, Parkville, Australia; 4 Division of Paediatric Critical Care Medicine, Department of Paediatrics, Beatrix Children’s Hospital, University Medical Center Groningen, Groningen, The Netherlands; 5 Critical Care, Anaesthesiology, Peri-Operative and Emergency Medicine, The University of Groningen, Groningen, The Netherlands; 6 Department of Anesthesiology and Intensive Care Medicine, University Medical Centre Schleswig–Holstein, Campus Kiel, Arnold-Heller-Straße 3, Haus R3, 24105 Kiel, Germany

**Keywords:** Infant, Preterm, Mechanical ventilation, Mechanical power, Lung mechanics, Ventilator-induced lung injury

## Abstract

**Background:**

Mechanical power is a major contributor to lung injury and mortality in adults receiving mechanical ventilation. Recent advances in our understanding of mechanical power have allowed the different mechanical components to be isolated. The preterm lung shares many of the same similarities that would indicate mechanical power may be relevant in this group. To date, the role of mechanical power in neonatal lung injury is unknown. We hypothesise that mechanical power maybe useful in expanding our understanding of preterm lung disease. Specifically, that mechanical power measures may account for gaps in knowledge in how lung injury is initiated.

**Hypothesis-generating data set:**

To provide a justification for our hypothesis, data in a repository at the Murdoch Children’s Research Institute, Melbourne (Australia) were re-analysed. 16 preterm lambs 124–127d gestation (term 145d) who received 90 min of standardised positive pressure ventilation from birth via a cuffed endotracheal tube were chosen as each was exposed to three distinct and clinically relevant respiratory states with unique mechanics. These were (1) the respiratory transition to air-breathing from an entirely fluid-filled lung (rapid aeration and fall in resistance); (2) commencement of tidal ventilation in an acutely surfactant-deficient state (low compliance) and (3) exogenous surfactant therapy (improved aeration and compliance). Total, tidal, resistive and elastic-dynamic mechanical power were calculated from the flow, pressure and volume signals (200 Hz) for each inflation.

**Results:**

All components of mechanical power behaved as expected for each state. Mechanical power increased during lung aeration from birth to 5 min, before again falling immediately after surfactant therapy. Before surfactant therapy tidal power contributed 70% of total mechanical power, and 53.7% after. The contribution of resistive power was greatest at birth, demonstrating the initial high respiratory system resistance at birth.

**Conclusions:**

In our hypothesis-generating dataset, changes in mechanical power were evident during clinically important states for the preterm lung, specifically transition to air-breathing, changes in aeration and surfactant administration. Future preclinical studies using ventilation strategies designed to highlight different types of lung injury, including volu-, baro- and ergotrauma, are needed to test our hypothesis.

## Introduction

Ventilation-induced lung injury (VILI) is a serious complication of mechanical ventilation (MV). VILI is a multifactorial process that describes the impact of biotrauma resulting from, amongst others, injurious volume, pressure and oxygen exposure during MV. Mechanical power (MP) describes the energy generated per minute by the mechanical process of tidal ventilation. Recently methods of calculating MP during pressure control ventilation have been described in children [1]. In adult patients with acute respiratory distress syndrome (ARDS), high MP has been shown to be independently associated with important clinical outcomes [2]. Specifically, increases in the dynamic-elastic component of MP (those related to driving pressure, tidal volume (*V*_*T*_) and respiratory rate) are associated with increased mortality [3].

Rates of preterm lung disease remain high, despite evidence-based strategies to reduce volutrauma, atelectasis and oxygen exposure [4]. It is widely accepted that there remains critical knowledge gaps in our understanding of the multifactorial factors that initiate preterm VILI [4]. The preterm lung shares many similarities with the ARDS lung, including low compliance, impaired oxygenation, surfactant deficiency, impaired ventilation–perfusion matching and upregulated inflammatory mediators [5]. Additionally, the preterm lung must support tidal ventilation before developmentally and structurally ready to do so. As the lung is an organ of motion, there is a strong biological rationale for the role of MP in preterm VILI. This hypothesis report considers whether MP may be useful in expanding our understanding of preterm VILI. We provide proof-of-concept re-analysis of existing data to define the components of mechanical power in preterm lambs undergoing a standardised MV strategy from birth during three distinctly different, but important, respiratory states with unique changes in lung mechanics.

## Hypothesis-generating data set

Post hoc re-analysis of pressure, flow and tidal volume data sampled at 200 Hz (Florian, Acutronic AG, Hirzel, Switzerland) was performed from 16 intubated and anaesthetised preterm lambs (cuffed endotracheal tube) who have been reported in detail before; mean (SD) weight 3.34 (0.47) kg, median (range) 126 (124–127) day gestation [6]. All lambs received a MV strategy for 90 min from birth that is the currently accepted lung protective approach to supporting the preterm lung; that is a static moderate PEEP, short inflation time, fast rate and low *V*_*T*_ using time cycled pressure limited ventilation with a targeted tidal volume mode. Specifically, PEEP was fixed at 8 cmH_2_O, tidal volume (*V*_*T*_) 5.5–8.0 ml/kg to maintain protocolised CO_2_ targets (40–60 mmHg), maximum peak inflating pressure (P_plat_) 40 cmH_2_O, rate 30–60 bpm and 0.4 s inflation time. MV was commenced at *V*_*T*_ 7 ml/kg and rate 60 bpm, and *V*_*T*_ increased if CO_2_ was above target. If CO_2_ was below target, *V*_*T*_ was first stepwise reduced. Rate was only reduced once *V*_*T*_ was 5.5 ml/kg. Exogenous surfactant (200 mg/kg poractant alfa) was administered at 10 min. Total, tidal, elastic-dynamic (elas) and resistive (res) MP were calculated for tidal inflations minutely to 5 min and then at 10, 15, 30 and 90 min using the geometric method [7]. That is, MP_total_ and MP_tidal_ were calculated from the area of the airway pressure–volume loop multiplied with 0.098*respiratory rate, with positive end-expiratory pressure (PEEP) subtracted for MP_tidal_ [8]. Respiratory system mechanics were determined by least squares fitting [9, 10], taking into account airway pressure (*P*_AW_), volume (*V*) and air flow (*V̇*) for all time points (*t*) of a breath to calculate respiratory system elastance (E_RS_) as well as both laminar and turbulent flow resistance (R_LAM_, R_TURB_) according to the equation [11]:

$$P_{{{\text{AW}}}} \left( t \right) \, = \, E_{{{\text{RS}}}} * \, V\left( t \right) \, + \, R_{{{\text{LAM}}}} * \, V\left( t \right) \, + \, R_{{{\text{TURB}}}} * \, V\left( t \right)^{{2}} + {\text{ PEEP}}{.}$$

The thereby determined value of *E*_RS_ was subsequently utilised for calculation of elastance pressure (P_ELAST_) from all volume samples of a breath according to the equation:

$$P_{{{\text{ELAST}}}} \left( t \right) \, = \, V\left( t \right) \, * \, E_{{{\text{RS}}}} .$$

Elastic power (MP_elas_) was then determined similar to MP_tidal_, by multiplying the area of the P_ELAST_-volume loop with 0.098* respiratory rate. This yielded the fraction of MP_tidal_ required to overcome elastic resistance of the respiratory system. Finally, resistive power (MP_res_) was calculated as the difference between MP_tidal_ and MP_elas_, if the fraction of MP_tidal_ not explained by the elastic properties of the respiratory system was due to resistance. The MP_res_:MP_elas_ was calculated to aid interpretation of the relative contribution of each at each time point. 2 lambs were excluded due to artefact in waveform signals.

**Fig. 1:**
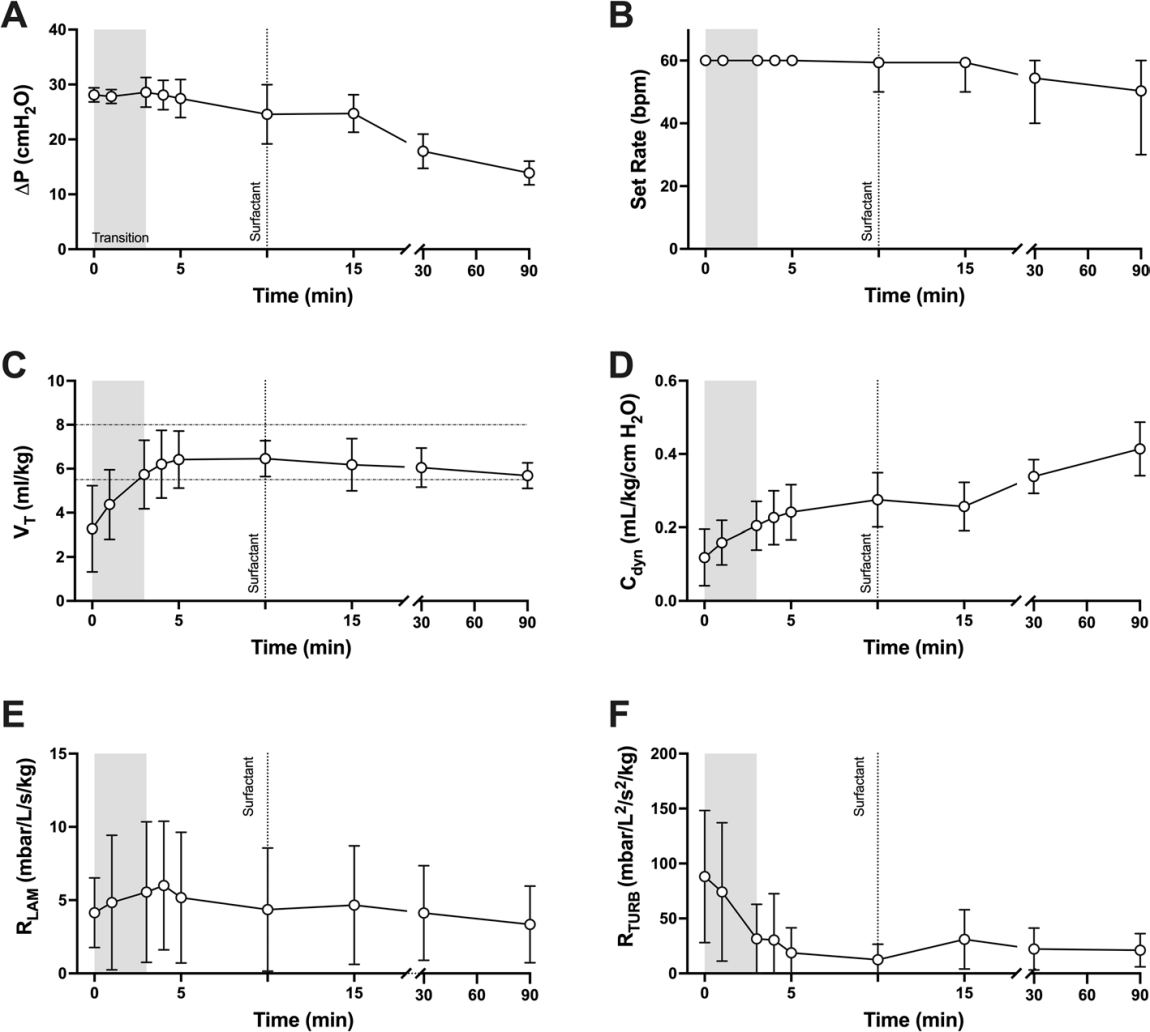
Behaviour of change in delivered pressure (Δ*P*; **A**), ventilator rate (bpm; inflations per min **B**), tidal volume (*V*_*T*_; **C**), dynamic compliance (*C*_dyn_; **D**), laminar (*R*_LAM_; **E**) and turbulent flow resistance (R_TURB_; **F**) during 90 min of standardised mechanical ventilation from birth (0 min) in 17 preterm lambs. The lung first underwent the transition (grey shading) from fluid-filled to aerated state during the first 3 min from birth; a period of falling lung resistance and increasing V_T_. Thereafter MV supported the aerated and surfactant-deficient preterm lung. Exogenous surfactant administered at 10 min (dotted vertical line) with resultant anticipated improvement in *C*_dyn_ and ventilator needs. **C** (*V*_*T*_) horizontal dash lines represent study protocol *V*_*T*_ target range. All data mean and SD, except ventilator rate (mean and range), and expressed against body weight where appropriate. Waveforms were excluded from analysis if artefact present (such as circuit fluid). *V*_*T*_ and *C*_dyn_ (both *p* < 0.0001) increased with time, whilst ventilator rate (*p* = 0.0015), Δ*P* (*p* < 0.0001) and R_TURB_ decreased (*p* = 0.004) and *R*_LAM_ (*p* = 0.32) was unchanged overall (all repeated measures two-way ANOVA)

During this 90-min period the lungs were first exposed to the respiratory transition at birth; a process of initial lung aeration from a fluid-filled state during which *R*_RS_ falls and dynamic compliance (*C*_dyn_) increases with sequential gains in aeration. Thereafter the structurally immature, poorly compliant preterm lung must support tidal ventilation, with exogenous surfactant designed to reduce this mechanical burden and increase *C*_dyn_. The delivered inflating pressure (ΔP; P_plat_-PEEP), ventilator rate, *V*_*T*_ and *C*_dyn_ data reflected these events; all *p* < 0.001, repeated-measure one-way ANOVA (Fig. 1A–D). R_LAM_ was not different over time (*p* = 0.32), whilst R_TURB_ quickly fell at birth (*p* = 0.004); Fig. 1E, F.

**Fig. 2:**
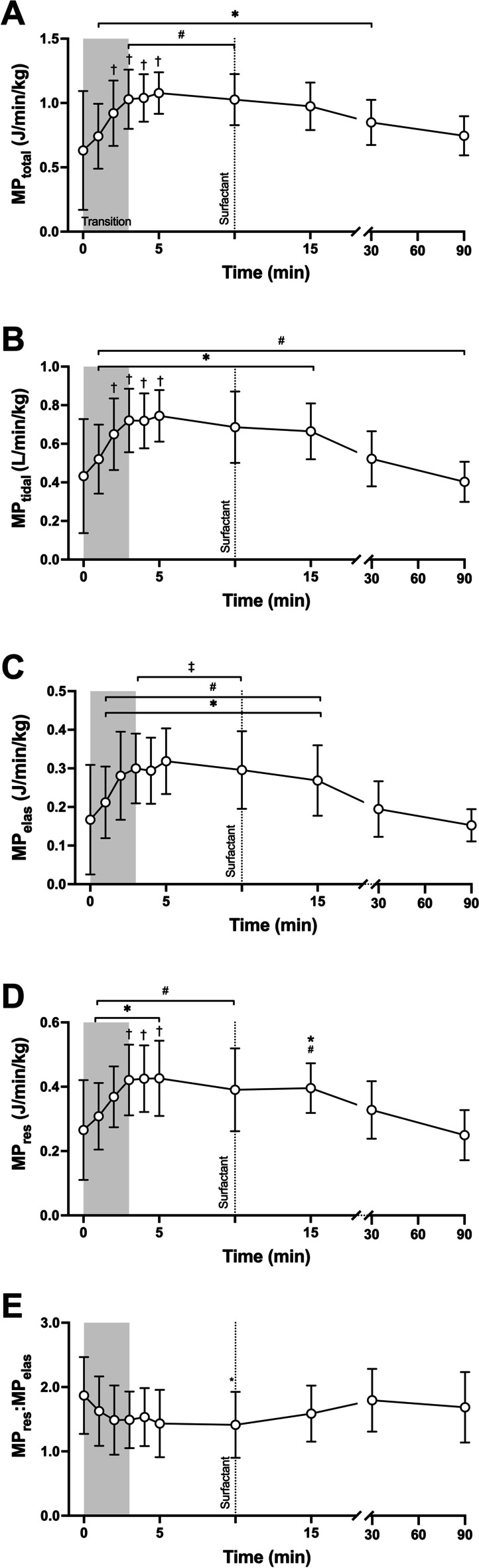
Behaviour of total mechanical power (MP_total_; **A**), mechanical power due to tidal ventilation (MP_tidal_; **B**), elastic-dynamic mechanical power (MP_elas_; **C**), resistive mechanical power (MP_res_; **D**) and ratio of MP_res_:MP_elas_ (**E**) during 90 min of standardised mechanical ventilation from birth as detailed in Fig. 1. Symbols and lines as per Fig. 1. All data mean and SD and expressed against body weight were appropriate. *p < 0.05 vs. birth (0 min), ^†^*p* < 0.05 vs. 1 min, ^#^*p* < 0.05 vs. 30 min, ^‡^*p* < 0.05 vs. 90 min (Tukey post-test, repeated-measure one-way ANOVA)

Overall, MP_total_, and the tidal, resistive and elastic-dynamic components, increased similarly during the first 5 min of MV at birth, with peak MP measures occurring at 3–5 min (the end of the respiratory transition); all *p* < 0.0001, repeated-measure one-way ANOVA (Fig. 2A–D). MP then stabilised until after surfactant administration, which resulted in a significant fall in all MP components. Tidal ventilation contributed to approximately 70% of MP_total_ during the respiratory transition. After surfactant therapy this fell to a mean (SD) 53.7 (6.3)% by 90 min (*p* < 0.0001). The contribution of MP_res_ to non-tidal MP was highest at birth, and rapidly fell to a baseline by 3 min (Fig. 2E); MP_res_:MP_elas_ mean (95%CI) difference between birth and minimum value at 10 min 0.46 (0.03,0.89), Tukey post-test.

## Discussion

To our knowledge, this is the first report of MP and energy transfer during MV in the preterm lung. As has been identified in the adult ARDS lung, this preliminary re-analysis suggests that MP may also provide useful, and more nuanced, insights into the direct consequences of MV in the evolution of preterm VILI. We intentionally selected a standardised and understood population of preterm lambs that underwent a range of clinical states associated with distinct changes in lung mechanics to consider our hypothesis [6]. Before MP can be used to compare different mechanical ventilation strategies in the preterm lung, it is first important to understand in which contexts it may be a useful addition to currently accepted measures (pressure *C*_dyn_, *V*_*T*_ and gas exchange).

The finding that MP decreased following surfactant administration is reassuring and expected, especially the large decrease in MP_tidal_. Surfactant reduces alveolar surface tension, thus improving *C*_dyn_ and the energy needed to move the lung. The observed fall in MP_res_, which was almost entirely related to laminar flow being the predominate component, is interesting. We postulate that this may reflect changes in chest wall mechanics and opening of small airways in the developmentally immature and atelectatic lung. MP_total_ and its tidal, elastance and resistive components provide the potential to quantify the energy impact beyond that of just *V*_*T*_ and *C*_dyn_ changes, including, R_RS_, respiratory rate, and indirectly PEEP. The impact of PEEP on lung mechanics is particularly important and often not considered in clinical practice. MP_total_ is directly influenced by PEEP, but MP_tidal_, MP_elas_ and MP_res_ are only impacted by PEEP if it effects elastance (MP_elas_), resistance (MP_res_) or both (MP_tidal_).

The most interesting, and potentially important, considerations occurred during the respiratory transition at birth. *R*_RS_ is known to fall during aeration at birth [12, 13], but we also observed an increase in MP_res_ during this period. This reflects the sequential gains in regional aeration that occur during the first few minutes at birth. Whilst the lung is in a highly resistive fluid-filled state at birth, there is no energy being transferred to the fluid-filled lung regions until aeration and then ventilation occurs in these regions [12]. This dynamic state of aeration and airway fluid clearance would explain the large fall in resistance due to turbulent rather than laminar flow. The risk of VILI begins during and once aeration commences, highlighting the need to avoid rapid lung aeration which are likely to transmit high energy potential compared to the ventilatory gains. This impact may be magnified as the lung gains aeration sequentially at birth [6, 12], with early aerating regions thus being exposed to the entire energy cost of mechanical ventilation. This may explain the increased regional injury noted following a sustained lung inflation at birth in preterm lambs [6], and provide insight into the unexpected higher early morbidity in a recent large trial of sustained inflation in apnoeic preterm infants [14].

## Future directions

From our preliminary explorations, we hypothesise that MP and its components can be used to understand the energetic impact, and thus ergotrauma potential, of the protective and injurious consequences of clinical decisions made in supporting the preterm lung. For example, whether an increase in rate with isocapnic decrease in *V*_*T*_ reduces or increases MP and alters VILI [15]. Determining whether (1) different MV strategies alter measures of MP, and (2) how these MV strategies impact VILI will be critical in understanding whether MP will add any further information than current MV measures. We propose that the preterm lamb provides an ideal experimental model for this. The preterm lamb shares biological and mechanical similarities with human infants. Unlike humans MV can be standardised to isolate different mechanical states, and more detailed instrumentation is possible (including cuffed endotracheal tubes). Importantly injury analysis can be conducted directly on lung tissue. The multifactorial nature of VILI, and the often inter-related impact of mechanical events and treatments, has limited separating the impact of different injury processes [6, 16, 17]. Recent advances in proteomics in the preterm lung have demonstrated the ability to isolate these processes, offering the potential to identify ergotrauma from the other ‘traumas’ (such as volutrauma) [18].

Simplified surrogate methods of calculating MP have been developed for both volume [3] and pressure-controlled ventilation [19, 20]. Such methods improve clinical translation. Similar simplified methods should be explored for the preterm lung.

## Conclusions

In our hypothesis-generating dataset, changes in mechanical power were evident during clinically important states for the preterm lung, specifically transition to air-breathing, changes in aeration and surfactant administration. In summary, MP may play an unrecognised role in understanding preterm VILI, particularly when *C*_dyn_ is not the predominant mechanical factor.

## Data Availability

All data, including raw data used for all figures and analysis, are available upon request from three months following article publication to researchers who provide a methodologically sound proposal, with approval by an independent review committee (“learned intermediary”). Proposals should be directed to david.tingay@mcri.edu.au to gain access. Data requestors will need to sign a data access or material transfer agreement approved by MCRI.
